# Urban-Rural Disparity of Breast Cancer and Socioeconomic Risk Factors in China

**DOI:** 10.1371/journal.pone.0117572

**Published:** 2015-02-17

**Authors:** Xufeng Fei, Jiaping Wu, Zhe Kong, George Christakos

**Affiliations:** 1 College of Environment and Resource Sciences, Zhejiang University, Hangzhou, China; 2 Institute of Islands and Coastal Ecosystems, Zhejiang University, Hangzhou, China; University of North Carolina School of Medicine, UNITED STATES

## Abstract

Breast cancer is one of the most commonly diagnosed cancers worldwide. The primary aim of this work is the study of breast cancer disparity among Chinese women in urban vs. rural regions and its associations with socioeconomic factors. Data on breast cancer incidence were obtained from the Chinese cancer registry annual report (2005–2009). The ten socioeconomic factors considered in this study were obtained from the national population 2000 census and the Chinese city/county statistical yearbooks. Student’s T test was used to assess disparities of female breast cancer and socioeconomic factors in urban vs. rural regions. Pearson correlation and ordinary least squares (OLS) models were employed to analyze the relationships between socioeconomic factors and cancer incidence. It was found that the breast cancer incidence was significantly higher in urban than in rural regions. Moreover, in urban regions, breast cancer incidence remained relatively stable, whereas in rural regions it displayed an annual percentage change (APC) of 8.55. Among the various socioeconomic factors considered, breast cancer incidence exhibited higher positive correlations with population density, percentage of non-agriculture population, and second industry output. On the other hand, the incidence was negatively correlated with the percentage of population employed in primary industry. Overall, it was observed that higher socioeconomic status would lead to a higher breast cancer incidence in China. When studying breast cancer etiology, special attention should be paid to environmental pollutants, especially endocrine disruptors produced during industrial activities. Lastly, the present work’s findings strongly recommend giving high priority to the development of a systematic nationwide breast cancer screening program for women in China; with sufficient participation, mammography screening can considerably reduce mortality among women.

## Introduction

Breast cancer is the most common malignancy tumor among women and the main cause of death among women cancer patients [1]. More than one million new breast cancer patients are diagnosed each year [2], and their distribution exhibits large geographical variations worldwide [3]. The age-standardized rates (ASR) in developed regions are three times higher than that in developing ones [1]. Female breast cancer incidence is highest in America, followed by northern and western Europe, the lowest being in Africa and Asia [4]. The ASR for female breast cancer in China was about 30/100,000 during 1998–2007 [5] with large differences between urban and rural regions [6, 7].

The associations between female breast cancer and early menarche, late age at first birth and late menopause, which affect the concentration of oestrogen in women body, have been confirmed in developed countries [8]. Exogenous hormones, such as hormone replacement therapy, were considered as breast cancer risk factors [9], but the relationships with other exogenous hormones, like oral contraceptives, may not be strong or consistent [10, 11], which is an issue that needs further exploration. Other possible risk factors for breast cancer include higher body mass index (BMI) [12], dietary habits [13, 14] and excessive alcohol consumption[15].

It is well known that there exist considerable regional disparities of cancer incidence, such as thyroid [16] and melanoma [17]. By comparing the distribution of cancer incidence between different geographical regions, one could obtain valuable clues concerning cancer pathology, and provide key insights to cancer preventive strategies and the optimization of medical resources allocation [2]. Breast cancer shows a large urban-rural disparity worldwide [18, 19]. The association between breast cancer and socioeconomic status has been well established [20, 21]. The socioeconomic factors and environmental determinants of breast cancer risk vary considerably (including diet, alcohol consumption, occupational exposure, physical exercise, and access to cancer screening and treatment) [22–24]. Accordingly, when studying “cancer incidence-socioeconomic status” associations, researchers usually are not limited to a single indicator, but rather include a combination of socioeconomic factors, such as multi-factorial socioeconomic index [17, 25] and European Deprivation Index (EDI) [26, 27]. However, due to the interaction between socioeconomic factors, one may not be able to adequately assess the relative influence of each factor in the above associations, which means that it is often difficult to rigorously explain cancer incidence mechanisms and offer useful policy advice (to remove social inequalities, distribute resources etc.).

In view of the above considerations, the objectives of the present observational study are to (1) analyze the temporal trend of female breast cancer incidence in China during the period 2005–2009, (2) detect incidence disparities among women living in urban vs. rural regions, and (3) determine associations between breast cancer incidence and socioeconomic factors, thus contributing to an improved understanding of possible cancer mechanisms and offering potentially useful health policy advise.

## Materials and Methods

### Data resources

**Fig 1:**
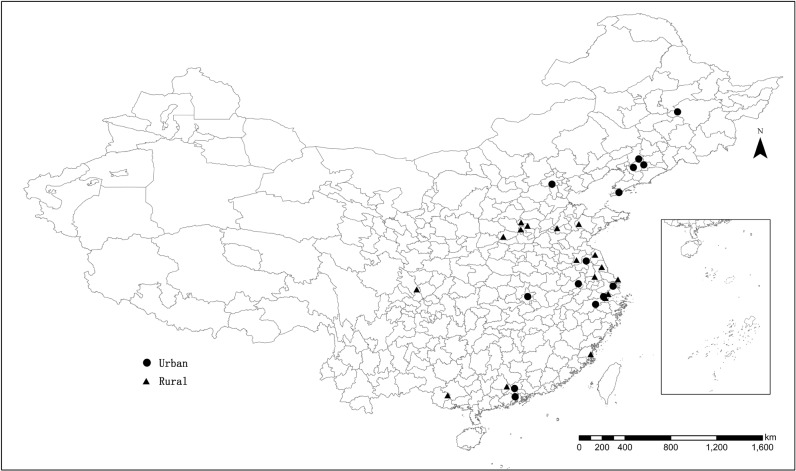
Geographic distribution of the cancer registries in China.

Data on age-adjusted female breast cancer incidence and on specified age group incidence during the years 2005–2009 were obtained from the Chinese cancer registry annual report [28]. All the cases were collected by local cancer registries following standard procedures to ensure data quality: when new cases occurred, cancer registration reporting cards were filled and submitted to cancer registries by local hospital staff for re-checking and correction, and the completeness and reliability of the collected data were subsequently checked by the National central cancer registry. Since the 2005 cancer registry annual report was lacking female breast cancer incidence data in Chinese cities, in the present work complete registry data were available for the years 2006–2009 in 32 cities. Among these 32 cities, Huaian with one district registry data was excluded from the analysis. Hence, 31 cities were used to analyze incidence at the city level, which were distributed in 19 provinces and municipalities; of these 31 cities, 14 were located in urban regions and 17 in rural regions. The total female population of these cities accounted for 5.53% of the entire female population at the national level (Fig. 1).

Many breast cancer determinants are difficult and expensive to evaluate directly [29]. For example, it would be costly, requiring considerable human resources to estimate the proportion of women at the city level who have ever received breast cancer screening. On the other hand, it is much easier to access the proxies of the determinants [29]. Accordingly, in this study ten socioeconomic variables were used as proxies of the breast cancer determinants: population density (10,000 people/km^2^), average number of years of education (years), illiteracy rate (%), unemployment rate (%), percentage of population employed in primary industry (%), in second industry (%), and in tertiary industry (%), percentage of non-agriculture population (%), primary industry output (100 million Yuan), and second industry output (100 million Yuan). These variables are proxies of environmental pollution, lifestyle, access to hormone replacement and oral contraceptive etc., which are direct determinants of breast cancer. S1 Fig. gives an outline of the relationships considered in this study between the breast cancer incidence, the direct determinants of this cancer, and the socioeconomic proxies of the determinants. One notices that, given the complexity of cancer mechanisms, explicit connections between determinants and mechanisms could not be defined in the present observational study. Instead, certain useful relationships between socioeconomic variables and cancer incidence can be derived in terms of OLS models. All the socioeconomic variables were obtained from the national population census (2000) [30], Chinese city statistical yearbooks (2006–2009) [31], and Chinese county statistical yearbooks (2006–2009) [32].

### Statistical analysis

The Student’s T test was used to assess disparities of female breast cancer and socioeconomic factors in urban vs. rural regions. Pearson correlation was used to analyze the relationships between socioeconomic factors and cancer incidence. Empirical OLS models were used to assess the predictive ability of the significant correlations between socioeconomic factors and breast cancer incidence, as follows:

y=ϑ 0+ ∑i=1kϑixi+ε (1)

where *y* is the predicted value of the dependent variable (female breast cancer incidence), ϑ_0_ is the intercept, *x*_i_ are the independent variables (socioeconomic factors), *k* is the number of independent variables, ϑ_i_ are empirical coefficients associated with *x*_i_, and *ɛ* is the error term.

According to the relevant *Q-Q* plots and histogram, the socioeconomic factors and breast cancer incidence were not normally distributed, so all the variables introduced into the OLS model were natural logarithm-transformed. Moreover, in order to avoid multicollinearity problems among socioeconomic factors, only one factor was considered at a time as the independent variable (for each OLS model). The above quantitative analysis was performed using SPSS 19.0 (IBM Corp., Armonk, NY), the significant tests were two-sided, and the threshold was set at 0.05.

Beyond empirical relationships, theoretical carcinogenesis models could be also included in future studies of breast cancer incidence among Chinese women, assuming that sufficient data and understanding are available, allowing the calculation of the corresponding model parameters, initial conditions, and other auxiliary variables. Generally, the use of sophisticated theoretical models of the cancer process is limited by the simultaneous needs (a) to establish a mathematical framework that adequately represents the carcinogenesis and (b) to maintain analytically or numerically tractable results [33]. A system of stochastic differential equations (SDE) of multistage carcinogenesis modeling is derived and solved to calculate tumor incidence. These SDE can be coupled with physiology-based pharmacokinetic models to link exposure biomarkers to population cancer risks.

## Results

**Fig 2:**
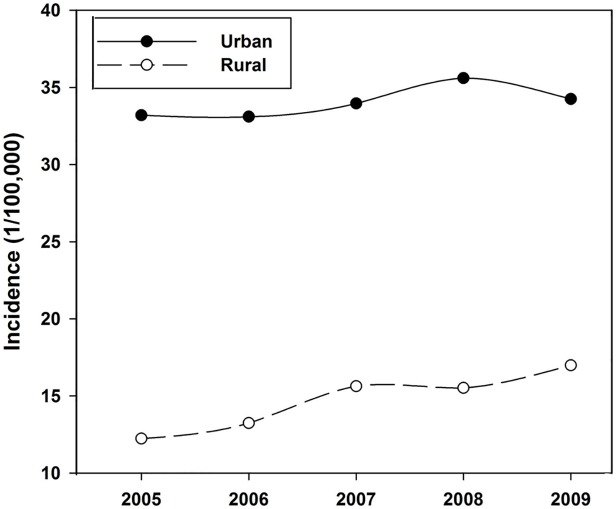
Female breast cancer incidence in Chinese urban *vs*. rural regions from 2005 to 2009.

**Fig 3:**
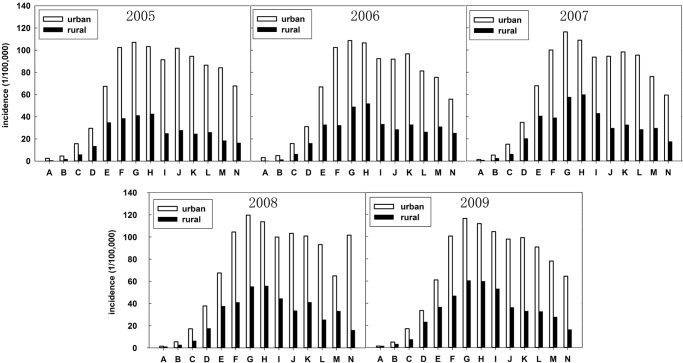
Female breast cancer incidence in different age groups during 2005–2009^a^. ^a^ Age groups: A: 0–24, B: 25–29, C: 30–34, D: 35–39, E: 40–44, F: 45–49, G: 50–54, H: 55–59, I: 60–64, J: 65–69, K: 70–74, L: 75–79, M: 80–84, N: > 85.

During the period 2005–2009, the female breast cancer incidence maintained a relative stable level in the Chinese registry areas: 28.32/100,000 (2005), 29.25/100,000 (2006), 29.87/100,000 (2007), 31.71/100,000 (2008), and 28.99/100,000 (2009), respectively. However, large differences were observed in female breast cancer incidence between urban and rural regions (Fig. 2). The incidence in urban regions (33.20/100,000 in 2005, 33.10/100,000 in 2006, 33.96/100,000 in 2007, 35.60/100,000 in 2008, and 34.25/100,000 in 2009, respectively) was about 2.3 times higher than that in rural regions (12.23/100,000 in 2005, 13.24/100,000 in 2006, 15.63/100,000 in 2007, 15.52/100,000 in 2008, and 16.98/100,000 in 2009, respectively). The incidence in urban regions showed no significant difference in these years, with the maximum (35.60/100,000 in 2008) being about 7.6% higher than the minimum (33.10/100,000 in 2006). In rural regions, however, the incidence increased fast, with the maximum incidence (16.98/100,000 in 2009) being about 38.8% higher than the minimum (12.23/100,000 in 2005), whereas incidence kept increasing with about 8.55 APC during 2005–2009. Fig. 3 shows the incidence distribution among different age groups. Almost no breast cancer case was detected among people 24 years old or younger. For adults 25–54 years old, the incidence presented an alarming increase, and reached the highest incidence for adults 50–54 years old, then it experienced a slight decrease for people older than 55 years old. More than 50% of the breast cancer cases during the period 2005–2009 were observed among people 40–64 years old.

Table 1 showed that there were large differences of female breast cancer incidence between different cities. Guangzhou (46.63/100,000), Dalian (43.37/100,000) and Anshan (41.64/100,000) had the highest average incidence. All the urban cities had breast cancer incidence higher than 20/100,000. Whereas, only three cities had incidence bigger than 20/100,000, among rural cities, i.e., Jiashan (29.23/100,000), Yangzhong (27.58/100,000) and Feichen (22.84/100,000). Fusui (6.99/100,000) had the lowest incidence among all cities. As one can observe, the highest incidence (Guangzhou) was about 6.7 times higher than the lowest incidence (Fusui). There were significantly differences (*P* < 0.01) between urban and rural cities during every year of the study (Table 2). At the same time, the ten socioeconomic factors considered in the study presented significant differences in urban vs. rural regions. In particular, population density, percentage of non-agriculture population, second industry output, percentage of population employed in tertiary industry, average years of education, unemployment rate, percentage of population employed in second industry, and primary industry output were significantly higher in urban regions. The reverse was the situation with the illiteracy rate and the population percentage employed in primary industry (Table 3).

**Table 1:** Female breast cancer incidence, urban vs. rural (U/R).

**City**	**U/R**	**Incidence (95% CI)**	**City**	**U/R**	**Incidence (95% CI)**
Jiashan	R	29.23 (23.94–36.30)	Guangzhou	U	46.63 (45.51–47.52)
Yangzhong	R	27.58 (23.02–32.52)	Dalian	U	43.37 (41.30–45.00)
Feichen	R	22.84 (16.88–28.81)	Anshan	U	41.64 (39.14–44.50)
Cixian	R	19.09 (15.49–23.06)	Beijing	U	39.84 (37.41–42.11)
Haimen	R	17.77 (14.98–20.55)	Shanghai	U	39.24 (37.98–40.26)
Hainin	R	17.01 (14.09–22.03)	Shengyang	U	36.55 (33.69–39.14)
Yangchen	R	16.35 (14.37–17.83)	Benxi	U	33.13 (30.39–35.27)
Dafeng	R	15.25 (13.17–16.43)	Wuhan	U	32.97 (31.96–33.88)
Linzhou	R	14.63 (12.32–16.85)	Harbin	U	30.94 (29.37–32.15)
Qidong	R	14.54 (13.06–16.68)	Jiaxing	U	30.89 (28.48–33.01)
Shexian	R	14.42 (11.49–17.33)	Hangzhou	U	29.99 (26.27–33.47)
Sihui	R	13.40 (12.11–15.54)	Suzhou	U	24.64(20.61–28.66)
Yanting	R	13.18 (9.95–14.88)	Maanshan	U	22.39 (18.62–26.15)
Linqu	R	13.05 (10.03–16.79)	Zhongshan	U	22.05 (19.63–23.97)
Changle	R	9.50 (8.74–10.28)			
Jianhu	R	8.61 (6.40–10.01)			
Fusui	R	6.99 (3.83–11.01)			

U: urban city, R: rural city, Incidence: 4-year average breast cancer incidence (1/100,000), CI: confidence interval.

**Table 2:** Comparison of breast cancer incidence in urban vs. rural regions (N = 31).

**Year**	**U/R**	**N**	**incidence**	**STD**	**95%CI**	***P***
2006	U	14	31.99	8.10	28.02–36.78	
	R	17	13.29	6.15	10.62–16.07	<0.001
2007	U	14	33.26	8.15	28.78–37.56	
	R	17	15.62	6.02	12.74–18.29	<0.001
2008	U	14	35.47	8.29	31.43–39.96	
	R	17	16.78	7.89	13.63–20.71	<0.001
2009	U	14	34.79	7.51	30.94–38.60	
	R	17	18.64	6.60	15.65–21.85	<0.001

U: urban city, R: rural city, N: the number of cities in urban/rural areas, incidence (1/100,000), STD: standard deviation, CI: confidence interval. *P*: significance of difference of incidence between urban and rural areas.

**Table 3:** Descriptive analysis of socioeconomic factors in urban vs. rural regions.

		**Urban**	**Rural**			**Urban**	**Rural**
PD	range	0.53–0.05	0.06–0.01	PEP	range	7.38–54.75	23.37–86.04
	mean	0.19	0.03		mean	30.02	59.67
	median	0.12	0.03		median	32.65	64.87
	T-test	P = 0.002**			T-test	P < 0.001**	
PNA	range	36.60–96.14	8.43–33.52	PU	range	2.51–15.40	1.07–11.17
	mean	77.16	18.73		mean	7.76	3.03
	median	85.71	17.95		median	8.13	2.42
	T-test	P < 0.001**			T-test	P < 0.001**	
SIO	range	96–5667	11–205	PES	range	17.15–61.38	4.06–51.47
	mean	1619	112		mean	36.43	23.41
	median	1300	101		median	33.64	22.60
	T-test	P = 0.004**			T-test	P = 0.011*	
PET	range	21.16–60.74	9.90–43.74	PI	range	5.03–17.38	6.12–18.22
	mean	33.55	16.92		mean	9.13	12.12
	median	30.20	13.54		median	8.57	11.36
	T-test	P < 0.001**			T-test	P = 0.022*	
EDU	range	6.56–9.97	6.45–7.50	PIO	range	2.43–123.94	4.22–41.44
	mean	8.17	7.00		mean	54.84	20.97
	median	8.25	7.00		median	49.06	21.46
	T-test	P < 0.001**			T-test	P = 0.008**	

PD: population density, PNA: percentage of non-agriculture population, SIO: second industry output, PET: percentage of population employed in tertiary industry, EDU: average years of education, PEP: percentage of population employed in primary industry, PU: percentage of unemployed population (unemployment rate), PES: percentage of population employed in second industry, PI: percentage of illiteracy (illiteracy rate), PIO: primary industry output.

* Correlation is significant at the 0.05 level (2-tailed).

** Correlation is significant at the 0.01 level (2-tailed).

We further classified the 31 cities into more/less developed ones according to the ten socioeconomic factors and the K-mean cluster. The results showed no difference to the urban/rural stratifications. All urban cities were classified as more developed cities, whereas the rural cities were classified as less developed ones (S1 Table). Moreover, the incidence difference between more developed and less developed cities was significant during every year (S2 Table).

Table 4.displays Pearson’s correlation coefficients between breast cancer incidence and various socioeconomic factors. In cities, the breast cancer incidence showed significant positive correlation with population density, percentage of non-agriculture population, second industry output, percentage of population employed in tertiary industry, average years of education, percentage of unemployed, and percentage of population employed in second industry. On the other hand, breast cancer incidence exhibited significant negative correlations with the percentage of population employed in primary industry. Cancer incidence showed insignificant correlation with the percentage of illiteracy and primary industry output. Consequently, we did not include these last two factors in the OLS regression model used in our analysis.

**Table 4:** Pearson’s correlation coefficients between socioeconomic factors and breast cancer incidence.

	**BC**
PD	0.800**
PNA	0.759**
SIO	0.745**
PET	0.681**
EDU	0.640**
PEP	-0.586**
PU	0.545**
PES	0.544**
PI	-0.288
PIO	0.267

PD: population density, PNA: percentage of non-agriculture population, SIO: second industry output, PET: percentage of population employed in tertiary industry, EDU: average years of education, PEP: percentage of population employed in primary industry, PU: percentage of unemployed (unemployment rate), PES: percentage of population employed in second industry, PI: percentage of illiteracy (illiteracy rate), PIO: primary industry output, BC: Breast cancer incidence.

** Correlation is significant at the 0.01 level (2-tailed).

There are significantly correlations among socioeconomic factors themselves (Table 5). In order to solve the problem of potential multi-collinearity among these factors and to compare the predictive ability of each factor, we used the factors one by one as independent variables into the OLS model (representing the association between socioeconomic factors and breast cancer incidence, Eq (1)). The results of OLS analysis are shown in Table 6. The predictive ability of each factor ranked as follow: population density (*R* ^2^ = 0.641), percentage of non-agriculture population (*R* ^2^ = 0.579), second industry output (*R* ^2^ = 0.555), percentage of population employed in tertiary industry (*R* ^2^ = 0.463), average years of education (*R* ^2^ = 0.409), percentage of population employed in primary industry (*R* ^2^ = 0.344), percentage of unemployed (*R* ^2^ = 0.296), percentage of population employed in second industry (*R* ^2^ = 0.296). With the exception of the percentage of population employed in primary industry, which is negatively associated with breast cancer incidence, the other seven factors are all positively associated with breast cancer incidence. Also, the contribution of each socioeconomic factor *x*_i_ on the incidence variable *y* is quantitatively expressed by the coefficients ϑ_i_ of the OLS model of Eq (1). Accordingly, in Table 6 we list numerical ϑ_i_-values for the ten socioeconomic variables, which express in quantitative terms the relative contribution of each socioeconomic factor on breast cancer incidence. The above findings and results could provide valuable insight as far as women’s health in China is concerned.

**Table 5:** Pearson’s correlation coefficients between socioeconomic factors.

	**PNA**	**SIO**	**PET**	**EDU**	**PEP**	**PU**	**PES**	**PI**	**PIO**
PD	0.713**	0.773**	0.760**	0.610**	-0.738**	0.570**	0.599**	-0.233	0.396*
PNA		0.690**	0.691**	0.739**	-0.508**	0.693**	0.420*	-0.419*	0.321
SIO			0.738**	0.698**	-0.764**	0.551**	0.678**	-0.373*	0.538**
PET				0.671**	-0.829**	0.644**	0.819**	-0.326	0.342
EDU					-0.560**	0.622**	0.243	-0.744**	0.458**
PEP						-0.513**	-0.742**	0.310	-0.421*
PU							0.396*	-0.362*	0.167
PES								-0.125	0.199
PI									-0.267

PD: population density, PNA: percentage of non-agriculture population, SIO: second industry output, PET: percentage of population employed in tertiary industry, EDU: education years (average), PEP: percentage of population employed in primary industry, PU: percentage of unemployed (unemployment rate), PES: percentage of population employed in second industry, PI: percentage of illiteracy (illiteracy rate), PIO: primary industry output, BC: Breast cancer incidence.

* Correlation is significant at the 0.05 level (2-tailed).

** Correlation is significant at the 0.01 level (2-tailed).

**Table 6:** Ordinary Least Squares (QLS) model for socioeconomic factors and breast cancer incidence.

**Explanatory variable**	**QLS model**	***R*^2^**
PD	BI = 4.10**+0.36PD**	0.641**
PNA	BI = 1.39**+0.47PNA**	0.576**
SIO	BI = 1.715**+0.24SIO**	0.555**
PET	BI = 1.00*+0.67PET**	0.463**
EDU	BI = -2.75*+2.89EDU**	0.409**
PEP	BI = 4.84**–0.48PEP**	0.344**
PU	BI = 2.56**+0.37PU**	0.296**
PES	BI = 1.68**+0.43PES**	0.296**

PD: population density, PNA: percentage of non-agriculture population, SIO: second industry output, PET: percentage of population employed in tertiary industry, EDU: average years of education, PEP: percentage of population employed in primary industry, PU: percentage of unemployed (unemployment rate), PES: percentage of population employed in second industry, PI: percentage of illiteracy (illiteracy rate), PIO: primary industry output, BC: Breast cancer incidence.

** Correlation is significant at the 0.01 level (2-tailed).

## Discussion

The present observational study found that women living in urban regions had a greater risk of breast cancer than those living in rural regions, a finding that is consistent with previous studies [18, 34]. A sound explanation for this urban vs. rural disparity was that urban women, especially those with health insurance, were more likely to have mammograms [35] that allowed early-stage breast cancer diagnosis [36]. On the contrary, women in rural regions were less likely to be provided adequate health services (diagnosis or treatment), and, hence, the difference in health care accessibility leads to higher cancer incidence recorded in urban regions than in rural ones. Moreover, the influence of western lifestyle resulted in higher BMI values and increasing alcohol consumption among the population [23], increasing accessibility to hormone replacement therapy and oral contraceptive [37], and higher exposure to xenoestrogens and other environment endocrine disruptors (EEDs) [34], which may be also related to higher breast cancer incidence in urban regions. Further studies are needed to examine these possible risk factors in sufficient detail.

It is worth noting that, unlike the United States, where breast cancer incidence keeps decreasing [20], the incidence remains relatively stable in Chinese urban regions (about 30/100,000 women). What’s worse, in Chinese rural regions the incidence showed an APC of about 8.55 from 2005 to 2009. Since the 1980s, large numbers of rural workers have migrated to urban cities seeking a better life. However, the majority of them were less educated and lacked special skills, so they had to occupy high exposure risk jobs in construction, textile industry etc. [38]. Thus, these migrant workers had a higher risk to develop breast cancer. Moreover, since these migrant workers are not official residents of those cities, most of them were excluded from an urban city’s medical and social security benefits [39]. When a migrant worker from a rural region was diagnosed with breast cancer, according to *Hukou* policy, the case was registered in the worker’s hometown and not in the urban city of its occupation, which maybe the explanation for the stable breast cancer incidence in urban regions and the dramatic incidence increase in rural regions.

Interestingly, the observed breast cancer incidence in China was lower compared to highly developed Western countries [4]. Previous studies also have pointed out that a relatively low percentage of Chinese women (21.7%) in urban regions have reported that they ever had received breast cancer mammographic screening. In rural regions this percentage was even lower (16.5%) [35]. Considering China’s high rate of development in recent decades, an increasing number of people can afford regular mammograms, in urban and in rural regions. It is an urgent matter to determine whether the increasing percentage of mammographic screening or some other environmental risk factors (EEDs etc.) are responsible for the fast cancer incidence increase observed in rural regions. The critical importance of the matter is emphasized by the fact that several observational studies broadly support trial findings that mammography screening substantially reduces breast cancer mortality [40]. In view of the above public health considerations, a systematic nationwide mammographic screening program should be developed with due urgency,especially for women older than 40 years.

Generally, cancer incidence increases with age [41]. In China, most cancer incidences (lung, liver, stomach, colon, etc.) are highest in the >70 age group (i.e., people older than 70 years) [28]. In particular, for female breast cancer incidence, the peak appeared in the 50–54 age groups. Breast cancer risk declines in the case of individuals older than 55 years. This phenomenon is different from that observed in the developed countries of the West whose incidence keeps a relative slow increase after age 50 [2]. China is a developing country, whose relative younger age structure of population would result in a flat or even negative breast cancer incidence curve after the menopause because of the increasing risks of occurrence in consecutive generations [2]. Moreover, breast cancer risk is strongly influenced by oestrogen concentration, and most women enter the menopause stage after the age of 55 years old (experiencing a declined concentration of body oestrogens), the diminishing levels of circulating oestrogen may result in the decreased breast cancer risk too [8, 42]. There also has an interesting phenomenon about incidence in different age groups (Fig. 3). The disease incidence in rural regions showed a decrease after the age of 65 years old. Two elements might contribute to this decrease: Firstly, rural women, especially older women, have a significantly smaller chance to get educated. A large percentage of them are illiterate, which leads to lower income, lower awareness of health state and lack of health insurance, directly or indirectly [43]. These older rural women can’t afford or are unwilling to seek treatment, resulting to a decreased disease incidence. Secondly, due to the tough living conditions in rural areas, the women, particular those of earlier generations, have a shorter life expectancy (less than 70 years), which would lead to the decreased incidence observed in older women.

There exist huge social-economic disparities between urban and rural areas. Urban cities are usually associated with higher industry output and population density, higher percentage of workers employed in second and tertiary industry, and higher percentage of non-agricultural population. People living in urban areas also get better education, resulting to a lower illiteracy prevalence, which is also among the findings of a previous study [43]. On the other hand, as the rural population is predominantly engaged in cultivating their own land, a higher percentage of the population is employed in primary industry so they are less likely to lose their job compared to their counterparts engaged in second/tertiary industry in urban areas. All these social-economic disparities are proxies of determinants of breast cancer incidence, as mentioned earlier. The analysis of the relationships between breast cancer incidence and socioeconomic factors may contribute to an improved understanding of the potential cancer mechanisms. Cancer is a chronic disease, the latency period varying from person to person. Early life exposures, such as smoking in adolescence, would lead to higher cancer risk in one’s future life [44]. The duration problem was too complex to discuss in the present study. Rather, we related breast cancer incidence to ten socioeconomic factors in a spatial perspective, assuming no big change of the social-economic disparities among cities in recent years and that these disparities would lead to different geographical distributions of breast cancer incidence.

In order to eliminate the multicollinearity effect among socioeconomic factors and gain a better insight into cancer mechanism, we analyzed these factors one-by-one in OLS model. Population density and percentage of non-agriculture population had the best predictive ability, exhibiting the highest correlation with breast cancer incidence. Being two comprehensive urbanization/development indices, the population density and the percentage of non-agriculture population may be linked to more breast cancer risk factors such as reproductive factors (later age at first birth, lower parity, etc.), easier access to hormone replacement therapy and oral contraceptive, higher exposure to xenoestrogens and other environmental endocrine disruptors, dietary habits (food with higher fat/calorie content), higher BMI etc. than the other eight socioeconomic indices which might be proxies of specific risk factor (for instance, average years of education is proxy of educational level) [8, 45]. As a result, these two socioeconomic factors showed higher positive correlation with breast cancer incidence. Meanwhile, due to historical and policy-related reasons, when a town is classified as urban area, all the people were regarded as urban population no matter what occupation they are engaged in. Population density might be a more powerful index for development level as there exists some misclassifications of non-agriculture population, which may contribute to a little higher predictive ability of population density than that of percentage of non-agriculture population.

Second industry output also had high predictive ability and was significantly correlated with breast cancer incidence. A reasonable interpretation for this result maybe that higher second industry output was not only a surrogate for higher urbanization and development level (which lead to higher access to mammography, hormone replacement therapy and oral contraceptive, western lifestyle and higher BMI [34, 45]), but it usually accompanied heavier industry pollution. Previous studies pointed out that resident proximity to industry and traffic facilities were associated with a higher breast cancer incidence [46]. Some other studies have found that environmental pollutants, especially xenoestrogenic environmental pollutants, had an impact on breast cancer [47, 48].

Percentage of population employed in primary industry was the only factor that was found to be negatively correlated with breast cancer. Higher percentage of population employed in primary industry usually occur in lower developed regions, and the women in those regions were lacking medical service [49], with higher parity and earlier age at first birth [2], and, therefore, they were less likely to be diagnosed with breast cancer. Other socioeconomic factors correlated with breast cancer incidence included: the average number of years of education and unemployed were usually used as indices for socioeconomic status [17, 25], the percentage of population employed in second/tertiary industry were common surrogates for cities’ level of development. These factors represented the difference between the breast cancer risk factors mentioned above (lifestyle, diet, reproductive factor, oestrogen and exogenous hormones, exposure to environmental pollutants) [22, 25, 34]. In general, better socioeconomic situations would be associated to higher breast cancer incidence.

There are certain issues to be noticed concerning the present study. Since only aggregated incidence and socioeconomic data were available, the relationships between breast cancer and socioeconomic factors could not be studied at the individual level. Moreover, due to lack of information concerning the percentage of mammographic screening at the city level, the contribution of increased percentage of screening to the rising disease incidence could not be assessed in rural regions, nor we could compare the breast cancer incidence according to different mammogram percentages among cities. Previous studies had argued that socioeconomic disparity may lead to health insurance inequalities, thus resulting in more late stage breast cancer cases and higher mortality in rural regions [20, 21]. Due to the existing privacy rules, we were able to obtain information neither about the diagnosis stage of a breast cancer patient nor about the specific death records. Hence, in this study we used mainstream statistics methods, Pearson correlation and OLS regression, to analyze the relationships between socioeconomic variables and breast cancer incidence. However, these mainstream methods lack the ability to assess the interaction between the socioeconomic variables, and we could not analyze the interaction using ANOVA (analysis of variance) or GLM (general linear model), because all the variables are numeric data. Moreover, due to the multicollinearity of the socioeconomic variables, it is inappropriate to combine them into a single regression model. Surely, socioeconomic factors are not the direct cause of breast cancer, rather the different levels of these factors represent disparities occurring in the actual breast cancer risk determinants (hormone level, reproductive factor, diet, lifestyle, physical exercise, etc.). Yet, although socioeconomic factors are not the cause of breast cancer, exploring the relationships between socioeconomic factors and breast cancer incidence could provide valuable insight into breast cancer pathology; whereas rigorous experimentation is subsequently needed to prove speculative cancer risk factors (e.g., different levels of socioeconomic factors represent disparities occurring in the actual breast cancer risk determinants, like hormone level, reproductive factor, diet, lifestyle, and physical exercise).

In sum, this work analyzed the urban-rural disparity of female breast cancer incidence and related the incidence to a number of socioeconomic factors. Breast cancer incidence stayed at a relative stable level in urban regions, whereas in rural ones it kept increasing significantly during 2005–2009. Considering the low mammograms participation rates among Chinese women, it is highly recommended that a nationwide breast cancer screening program be developed for public health purposes, the present observational study supporting the view that, with sufficient participation, mammography screening can substantially reduce breast cancer mortality among Chinese women. Overall, it was observed that higher socioeconomic status would lead to a higher breast cancer incidence in China. Population density, percentage of non-agriculture population and secondary industry output were significantly correlated with breast cancer incidence, which may imply that not only the disparities between urban and rural regions (lifestyle, diet, reproductive factors etc.), but also the environmental pollutants generated during industrial activities can have an impact on breast cancer incidence.

## Supporting Information

S1 Fig Outline of the relationships between breast cancer incidence, direct disease determinants, and socioeconomic proxies to determinants. Given the complexity of cancer mechanisms, explicit connections between determinants and incidence could not be defined (which is indicated by “?”). Instead, relationships between socioeconomic variables (which are known to be linked to disease determinants as indicated by “√”) and the breast cancer incidence can be derived in terms of OLS models.
(TIF)

S1 Table Female breast cancer incidence in more/less developed cities. (DOCX)

S2 Table Comparison of breast cancer incidence in more *vs*. less developed regions (N = 31). (DOCX)
