# Supplementary figures and images for: Urban-Rural Disparity of Breast Cancer and Socioeconomic Risk Factors in China
Source: PLoS One. 2015 Feb 17;10(2):e0117572. doi: 10.1371/journal.pone.0117572 (PMC4331531; doi:10.1371/journal.pone.0117572)

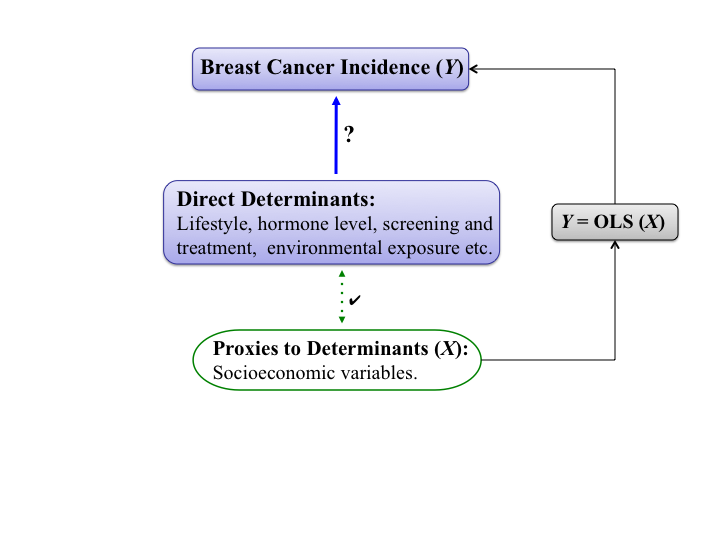

Supplement: S1 Fig — Given the complexity of cancer mechanisms, explicit connections between determinants and incidence could not be defined (which is indicated by “?”). Instead, relationships between socioeconomic variables (which are known to be linked to disease determinants as indicated by “√”) and the breast cancer incidence can be derived in terms of OLS models. (TIF) [file pone.0117572.s001.tif]
