# Supplementary material for: Urban-Rural Disparity of Breast Cancer and Socioeconomic Risk Factors in China
Source: PLoS One. 2015 Feb 17;10(2):e0117572. doi: 10.1371/journal.pone.0117572 (PMC4331531; doi:10.1371/journal.pone.0117572)
Supplement: S1 Table — (DOCX) [file pone.0117572.s002.docx]

Table S1: Female breast cancer incidence in more/less developed cities.

| City | M/L | Incidence (95% CI) | City | M/L | Incidence (95% CI) |
| --- | --- | --- | --- | --- | --- |
| Jiashan | L | 29.23 (23.94-36.30) | Guangzhou | M | 46.63 (45.51-47.52) |
| Yangzhong | L | 27.58 (23.02-32.52) | Dalian | M | 43.37 (41.30-45.00) |
| Feichen | L | 22.84 (16.88-28.81) | Anshan | M | 41.64 (39.14-44.50) |
| Cixian | L | 19.09 (15.49-23.06) | Beijing | M | 39.84 (37.41-42.11) |
| Haimen | L | 17.77 (14.98-20.55) | Shanghai | M | 39.24 (37.98-40.26) |
| Hainin | L | 17.01 (14.09-22.03) | Shengyang | M | 36.55 (33.69-39.14) |
| Yangchen | L | 16.35 (14.37-17.83) | Benxi | M | 33.13 (30.39-35.27) |
| Dafeng | L | 15.25 (13.17-16.43) | Wuhan | M | 32.97 (31.96-33.88) |
| Linzhou | L | 14.63 (12.32-16.85) | Harbin | M | 30.94 (29.37-32.15) |
| Qidong | L | 14.54 (13.06-16.68) | Jiaxing | M | 30.89 (28.48-33.01) |
| Shexian | L | 14.42 (11.49-17.33) | Hangzhou | M | 29.99 (26.27-33.47) |
| Sihui | L | 13.40 (12.11-15.54) | Suzhou | M | 24.64(20.61-28.66) |
| Yanting | L | 13.18 (9.95-14.88) | Maanshan | M | 22.39 (18.62-26.15) |
| Linqu | L | 13.05 (10.03-16.79) | Zhongshan | M | 22.05 (19.63-23.97) |
| Changle | L | 9.50 (8.74-10.28) |  |  |  |
| Jianhu | L | 8.61 (6.40-10.01) |  |  |  |
| Fusui | L | 6.99 (3.83-11.01) |  |  |  |

M: more developed city, L: less developed city, Incidence: 4-year average breast cancer incidence (1/100,000),

CI: confidence interval.
