# Supplementary material for: Urban-Rural Disparity of Breast Cancer and Socioeconomic Risk Factors in China
Source: PLoS One. 2015 Feb 17;10(2):e0117572. doi: 10.1371/journal.pone.0117572 (PMC4331531; doi:10.1371/journal.pone.0117572)
Supplement: S2 Table — (DOCX) [file pone.0117572.s003.docx]

Table S2: Comparison of breast cancer incidence in more *vs.* less developed regions (N=31)

| Year | M/L | N | incidence | STD | 95%CI | *P* |
| --- | --- | --- | --- | --- | --- | --- |
| 2006 | M | 14 | 31.99 | 8.10 | 28.02-36.78 |  |
|  | L | 17 | 13.29 | 6.15 | 10.62-16.07 | <0.001 |
| 2007 | M | 14 | 33.26 | 8.15 | 28.78-37.56 |  |
|  | L | 17 | 15.62 | 6.02 | 12.74-18.29 | <0.001 |
| 2008 | M | 14 | 35.47 | 8.29 | 31.43-39.96 |  |
|  | L | 17 | 16.78 | 7.89 | 13.63-20.71 | <0.001 |
| 2009 | M | 14 | 34.79 | 7.51 | 30.94-38.60 |  |
|  | L | 17 | 18.64 | 6.60 | 15.65-21.85 | <0.001 |

M: more developed city, L: less developed city, N: the number of cities in more/less developed areas, incidence (1/100,000), STD: standard deviation, CI: confidence interval. *P*: significance of difference of incidence between urban and rural areas.
